# Long-term survival trends in patients with unresectable stage III non-small cell lung cancer receiving chemotherapy and radiation therapy: a SEER cancer registry analysis

**DOI:** 10.1186/s12885-020-06734-3

**Published:** 2020-04-05

**Authors:** Ryan N. Hansen, Yiduo Zhang, Brian Seal, Kellie Ryan, Candice Yong, Annie Darilay, Scott D. Ramsey

**Affiliations:** 1 School of Pharmacy, University of Washington, 1959 NE Pacific, H-375, Box 357630, Seattle, WA 98195 USA; 2 AstraZeneca, One Medimmune Way, Gaithersburg, MD 20878 USA; 3 Fred Hutchinson Cancer Research Center, 1100 Fairview Avenue North, M3-B232, Seattle, WA 98109 USA

**Keywords:** Chemotherapy, Non-small cell lung cancer, Overall survival, Radiation therapy, Registry

## Abstract

**Background:**

To evaluate the value of new therapies for non-small cell lung cancer (NSCLC), it is necessary to understand overall survival (OS) rates associated with previous standard therapies and how these rates have evolved over time.

**Methods:**

We retrospectively analyzed data from patients enrolled in the Surveillance, Epidemiology, and End Results (SEER) cancer registry. Adults with unresectable, stage III NSCLC treated with chemoradiotherapy were grouped by diagnosis year (2000–2002; 2003–2005; 2006–2008; 2009–2011; 2012–2013). The primary endpoint was OS (data cut-off, December 31, 2014), estimated using the Kaplan–Meier estimator. Temporal survival-trend significance was tested using a two-sided log-rank trend test.

**Results:**

Of 12,865 eligible patients, 59.1% were male, 59.9% had stage IIIB disease, and 62.7% had non-squamous histology. Median age at diagnosis was 67 years. Overall, 10,899 (84.7%) patients died and 1966 (15.3%) were censored/lost to follow-up. Median follow-up (95% confidence interval [CI]) was 80 (77–82) months; median OS (95% CI) was 15 (15–16) months; 1- and 3-year survival probabilities (95% CI) were 57.7% (56.9–58.6) and 24.1% (23.3–24.8), respectively. Stratification by diagnosis year showed consistent improvements in survival over time (*p* < 0.0001 for trend). Median OS was 12, 14, 15, 18, and 19 months in successive cohorts.

**Conclusions:**

OS in patients diagnosed with unresectable, stage III NSCLC between 2003 and 2013 was consistent with that from clinical studies of sequential/concurrent chemoradiotherapy. Despite improvement over time, median OS was < 2 years and mortality remained high during the first year post-diagnosis.

## Background

Non-small cell lung cancer (NSCLC) comprises 85–90% of all lung cancer cases and is a leading cause of cancer death globally [1]. Approximately 30% of patients with NSCLC present with stage III, locally advanced disease, most of whom (with stage IIIB disease) have unresectable tumors [2]. The definition of “unresectable” can be subjective, depending on tumor size/location, the experience/judgment of the thoracic surgeon, and the fitness level of the patient [3]. The treatment goal for patients with unresectable disease is curative intent through eradicating visible intrathoracic disease, preventing local recurrence, and reducing the incidence of distant extrathoracic metastases. Although the goal is cure for unresectable stage III disease, this is achieved infrequently with current treatments, with a 5-year relative survival rate of 29.7% [4].

Over the last 40 years, there has been only modest progress in the therapeutic management of unresectable stage III NSCLC. In the 1980s, only radiation therapy was available, and median overall survival (OS) was approximately 10 months. By the 1990s, addition of sequential chemotherapy increased median OS to ~ 14 months [5], and when concurrent chemoradiotherapy (CRT) was established in the 2000s, median OS increased to 18 months [6].

The current standards of care for patients with unresectable stage III NSCLC include definitive platinum-based CRT followed by targeted immunotherapy with durvalumab (PACIFIC regimen) [7–9], which was approved in February 2018 in the US (patients whose disease has not progressed following platinum-based cCRT) and September 2018 in the EU (patients with tumors that express PD-L1 on ≥1% of tumor cells whose disease has not progressed following platinum-based CRT) [10, 11]. The aim of our analysis was to understand the impact that previous standard treatments had on OS in order to help determine the value of novel therapies. Therefore, we retrospectively analyzed OS data from patients with unresectable stage III NSCLC enrolled in the Surveillance, Epidemiology, and End Results (SEER) cancer registry [12], in the era before the approval of targeted immunotherapies.

## Methods

### Study design and patients

The SEER cancer registry collects and publishes data from various population-based cancer registries covering approximately 34% of the US population [12]. Our analysis population comprised patients aged ≥18 years diagnosed between 2000 and 2013 with unresectable stage III NSCLC (American Joint Committee on Cancer [AJCC] stage 3rd edition for cases diagnosed from 2000 to 2003 and AJCC stage 6th edition for cases diagnosed after 2003). These dates were chosen to reflect the timeframe in which CRT was incorporated into standard practice and to allow enough follow-up time for survival to be measured in each cohort. Eligible patients had received CRT; whether chemotherapy was concurrent with or sequential to radiotherapy was not recorded in the registry. Lung primary tumor site was identified by site codes C340, C341, C342, C343, C344, C345, C346, C347, C348, or C349; and histology by ICD-O-3 codes 8140, 8070, 8046, 8250, 8560, 8071, 8012, 8480, 8072, 8481, 8490, 8570, 8255, 8550, or 8260. Initial treatment following diagnosis was identified by binary indicators: surgery of primary site = 00; radiation treatment = 1; and chemotherapy received = 1; exact treatment dates were not included in the registry. We used a lack of recording of surgical resection, as denoted by SEER records, as a proxy for unresectability. Patients were grouped into cohorts by year of diagnosis: 2000–2002; 2003–2005; 2006–2008; 2009–2011; and 2012–2013. The study was Institution Review Board-approved by the Human Subjects Division at the University of Washington. The primary endpoint was OS, measured from diagnosis of unresectable stage III NSCLC to death from any cause, censoring (patient lost to follow-up in the registry), or data cut-off (December 31, 2014).

### Statistical analysis

Demographic and clinical characteristics were summarized for the total study population and by each diagnosis-year cohort. Median follow-up was calculated using the reverse Kaplan–Meier (K–M) method, with indicator variables reversed for death and censored events [13]. OS was estimated using the K–M method (SAS proc. lifetest) for the total population and each cohort. Survival curves were estimated for each cohort, with median OS calculated for both the total study population and each cohort, with Hall–Wellner 95% confidence bands. The two-sided log-rank trend test was used to test for a linear trend in the survival curves of the cohorts. One-year and 3-year survival probabilities were also calculated for the total study population and each cohort. To understand the change in mortality hazard as patients survived each subsequent year post-CRT, the conditional proportion of patients surviving each of the first 5 years post-diagnosis was determined for each diagnosis-year cohort. We used SAS 9.3 software for data management and statistical analyses.

## Results

### Patients

The SEER cancer registry included 239,602 patients diagnosed with lung cancer during the period 2000–2013, of whom 33,507 patients were diagnosed with unresectable stage III NSCLC. Overall, 12,865 patients were eligible for inclusion, having received radiation therapy and chemotherapy as their initial treatment.

Most patients were male (59.1%) and had stage IIIB disease (59.9%) and non-squamous histology (62.7%); median age at diagnosis was 67 years (Table 1). Race proportions (Asian, 3.5–4.3%; Black, 11.8–15.9%; White, 76.6–80.6%) and median age at diagnosis (66.0–68.0 years) were similar across cohorts. The proportion of patients diagnosed each year was also distributed evenly, with every year contributing 6.7–7.8% of the total sample, except for the year 2000, which contributed only 4.9%. There was a numerical trend towards earlier diagnosis over time, with stage IIIA NSCLC diagnosed in 33.2% of the 2000–2002 cohort, versus 46.5% of the 2012–2013 cohort.

**Table 1:** Demographic and clinical characteristics of the overall study population and by year of diagnosis cohort

	Total population (***N*** = 12,865)	Year of diagnosis cohort				
		2000–2002 (***n*** = 2380)	2003–2005 (***n*** = 2808)	2006–2008 (***n*** = 2926)	2009–2011 (***n*** = 2881)	2012–2013 (***n*** = 1870)
Age at diagnosis (years), median (IQR)	67 (59–74)	66 (58–73)	67 (59–74)	67 (59–74)	67 (59–75)	68 (60–74)
Male, *n* (%)	7599 (59.1)	1448 (60.8)	1703 (60.6)	1731 (59.2)	1666 (57.8)	1051 (56.2)
Race/ethnicity, *n* (%)						
Asian	503 (3.9)	82 (3.5)	111 (4.0)	120 (4.1)	125 (4.3)	65 (3.5)
Black	1734 (13.5)	316 (13.3)	330 (11.8)	413 (14.1)	377 (13.1)	298 (15.9)
White	10,168 (79.0)	1891 (79.5)	2264 (80.6)	2300 (78.6)	2281 (79.2)	1432 (76.6)
Other	453 (3.5)	89 (3.7)	101 (3.6)	92 (3.1)	97 (3.4)	74 (4.0)
Unknown	7 (0.1)	2 (0.1)	2 (0.1)	1 (0.0)	1 (0.0)	1 (0.1)
NSCLC stage, *n* (%)						
Stage IIIA	5159 (40.1)	791 (33.2)	1047 (37.3)	1198 (40.9)	1254 (43.5)	869 (46.5)
Stage IIIB	7706 (59.9)	1589 (66.8)	1761 (62.7)	1728 (59.1)	1627 (56.5)	1001 (53.5)
Histology, *n* (%) Squamous Non-squamous	4797 (37.3) 8068 (62.7)	876 (36.8) 1504 (63.2)	908 (32.3) 1900 (67.7)	970 (33.2) 1956 (66.9)	1200 (41.7) 1681 (58.4)	843 (45.1) 1027 (54.9)
Year of diagnosis, *n* (%)^*^						
2000	631 (4.9)	631 (26.5)				
2001	865 (6.7)	865 (36.3)				
2002	884 (6.9)	884 (37.1)				
2003	872 (6.8)		872 (31.1)			
2004	984 (7.7)		984 (35.0)			
2005	952 (7.4)		952 (33.9)			
2006	933 (7.3)			933 (31.9)		
2007	988 (7.7)			988 (33.8)		
2008	1005 (7.8)			1005 (34.3)		
2009	962 (7.5)				962 (33.4)	
2010	931 (7.2)				931 (32.3)	
2011	988 (7.7)				988 (34.3)	
2012	933 (7.3)					933 (49.9)
2013	937 (7.3)					937 (50.1)

^*^Percentage shown is based on total number of patients recruited overall or in each cohort, as applicable

*Abbreviations: IQR* Interquartile range; *NSCLC* Non-small cell lung cancer

### Overall survival

**Fig. 1:**
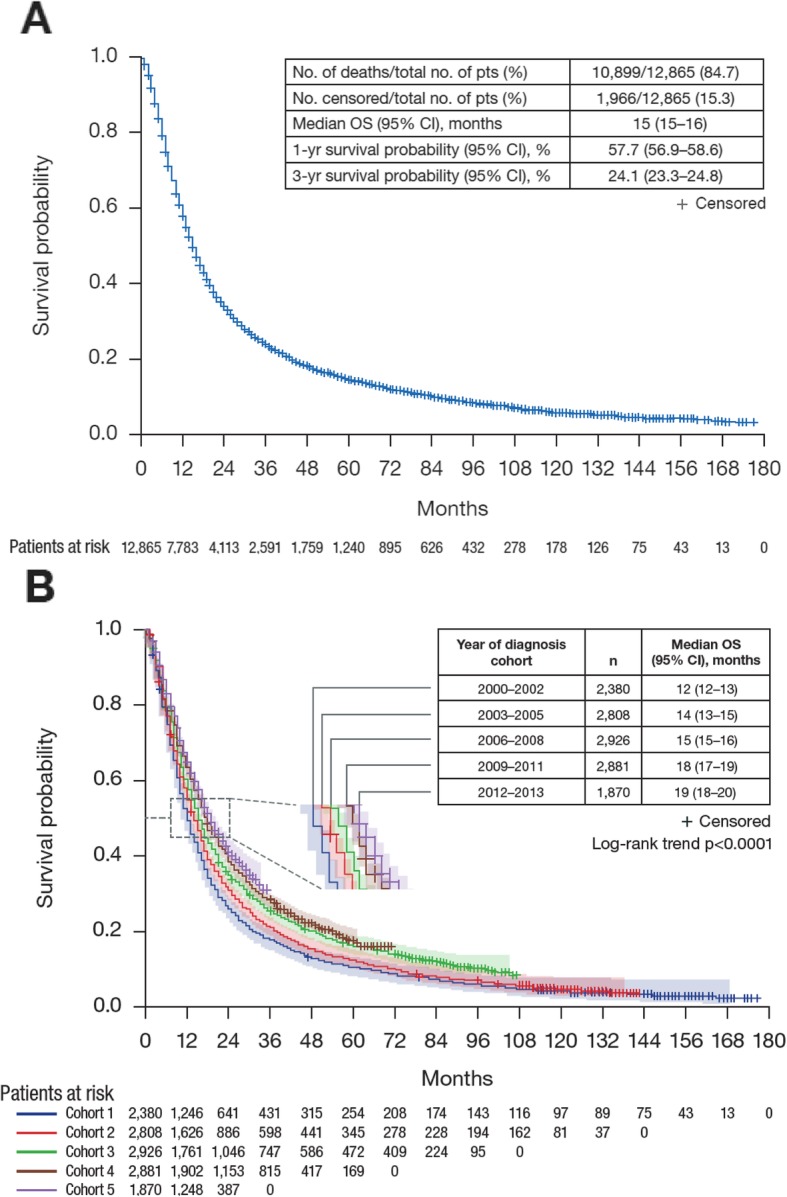
Kaplan–Meier curves of overall survival with number of patients at risk (A) in the total study population and (B) by year of diagnosis cohort. **A***Abbreviations: CI* Confidence interval; *no.* Number; *OS* Overall survival; *pts* Patients; *yr* Year. **B** Shading above and below survival curves represents 95% CIs. *Abbreviations: CI* Confidence interval; *OS* Overall survival

In total, 10,899 of 12,865 patients (84.7%) died and 1966 patients (15.3%) were censored or lost to follow-up during the study period. Median follow-up (95% confidence interval [CI]) was 80 (77–82) months in the overall population, and 158 (154–160), 125 (120–128), 88 (86–91), 53 (52–55), and 23 (23–24) months, respectively, in each successive cohort. Median OS (95% CI) for the total population was 15 (15–16) months, with 1- and 3-year survival probabilities (95% CI) of 57.7% (56.9–58.6) and 24.1% (23.3–24.8), respectively (Fig. 1a).

When stratified by year of diagnosis cohort, OS improved significantly over time (*p* < 0.0001 for trend; Fig. 1b and Table 2). Median OS increased from 12 months in the 2000–2002 cohort to 19 months in the 2012–2013 cohort, and respective 1-year survival rates increased from 49 to 65%. Across cohorts, the conditional 1-year survival probability (i.e. conditional probability of surviving another year) was similar between 0 and 1 year but increased after surviving 2 years from diagnosis. For the first four cohorts, where 5-year follow-up was possible, the conditional 1-year survival probability increased between 1 year and 4 years by ≥17%, with the probability ranging from 79 to 82% after surviving 4 years from diagnosis (Table 2).

**Table 2:** Median overall survival, survival rates, and conditional 1-year survival probabilities, by year of diagnosis cohort

	Year of diagnosis cohort				
	2000–2002 (***n*** = 2380)	2003–2005 (***n*** = 2808)	2006–2008 (***n*** = 2926)	2009–2011 (***n*** = 2881)	2012–2013 (***n*** = 1870)
Deaths, *n* (%)	2308 (97.0)	2676 (95.3)	2591 (88.6)	2287 (79.4)	1037 (55.4)
Patients censored, *n* (%)	72 (3.0)	132 (4.7)	335 (11.4)	594 (20.6)	833 (44.5)
Median OS (95% CI), months	12 (12–13)	14 (13–15)	15 (15–16)	18 (17–19)	19 (18–20)
1-year survival (95% CI), %	49.2 (47.2–51.2)	54.9 (53.1–56.8)	57.4 (55.6–59.2)	63.3 (61.5–65.0)	64.5 (62.5–66.8)
3-year survival (95% CI), %	17.8 (16.2–19.3)	20.8 (19.2–22.3)	25.3 (23.8–26.9)	28.0 (26.3–29.6)	–
5-year survival (95% CI), %	10.6 (9.4–11.8)	12.3 (11.0–13.4)	16.2 (14.9–17.5)	17.3 (15.7–18.9)	–
10-year survival (95% CI), %	4.1 (3.3–4.9)	4.8 (4.0–5.6)	_	–	–
Conditional 1-year survival probability (95% CI) after surviving …, %					
Year 0	49.2 (47.2–51.2)	54.9 (53.1–56.8)	57.4 (55.6–59.2)	63.3 (61.5–65.0)	64.5 (62.5–66.8)
Year 1^*^	52.7 (49.9–55.6)	56.1 (53.7–58.6)	60.5 (58.2–62.8)	61.2 (59.0–63.4)	63.4 (60.1–66.7)
Year 2^†^	68.4 (68.0–68.8)	67.3 (64.1–70.4)	73.0 (70.3–75.7)	72.2 (69.6–74.9)	–
Year 3^‡^	73.0 (68.7–77.2)	73.8 (70.3–77.4)	78.4 (75.4–81.4)	78.3 (75.1–81.4)	–
Year 4^§^	81.8 (77.4–86.1)	80.0 (76.2–83.7)	81.6 (78.4–84.8)	79.1 (74.3–83.8)	–

^*^1-year survival probability conditional on surviving year 1

^†^1-year survival probability conditional on surviving year 2

^‡^1-year survival probability conditional on surviving year 3

^§^1-year survival probability conditional on surviving year 4

*Abbreviations: CI* Confidence interval; *OS* Overall survival

## Discussion

This large observational study showed that OS in “real-world” patients diagnosed with unresectable stage III NSCLC between 2003 and 2013 was consistent with that reported in clinical trials of concurrent CRT [6]. OS increased significantly in successive diagnosis-year cohorts, consistent with findings from an earlier observational study of SEER registry data, which identified improvements in 5-year relative survival for all stages (separately) of NSCLC between 1988 and 2008 [14]. Reasons underlying these improvements are unclear, but could include successive increases in the adoption of concurrent CRT as a standard of care following its introduction in the early 2000s; choice of chemotherapy regimen; improvements in clinical management and palliative treatment outcomes including use of targeted therapies such as EGFR, VEGF and ALK inhibitors in later disease stages; and advances in chemotherapy and radiotherapy delivery. Another potential reason for the improvement relates to advances in imaging including more widespread use of PET/CT [15, 16], resulting in fewer patients with stage IV/metastases being included erroneously, or the increasing proportion of patients diagnosed at early stage (stage IIIA). Increases in staging accuracy may also have resulted in better patient selection and treatment choices.

Despite improvement over time, median OS for the total population was < 2 years and mortality risk remained high during the first year post-diagnosis, suggesting local control and distant metastases prevention remain a major challenge. Nevertheless, since unresectable stage III disease is a curative setting, it was perhaps unsurprising that survival benefits occurred after patients had survived the first 2 years post-diagnosis. This also suggests that the largest opportunity to improve long-term survival occurs during the first 2 years post-CRT. Indeed, after year 2, the conditional survival probability did not differ markedly between diagnosis-year cohorts. Although the time period for our analysis did not cover the introduction of durvalumab for patients with unresectable stage III NSCLC, we acknowledge that the PACIFIC regimen has since led to improvements in OS (12-, 24- and 36-month OS [durvalumab vs placebo]: 83.1% vs 74.6%, 66.3% vs 55.3%, and 57.0% vs 43.5%, respectively) and PFS (median 16.8 months vs 5.6 months, respectively), [8, 9, 17] helping to address the unmet needs of this population.

The study was limited by how data are recorded in SEER – it does not track performance status, whether chemotherapy was concurrent/sequential to radiation therapy, or whether treatment was completed by patients, which could each have affected outcomes. In addition, for the 2012–2013 cohort, only 2-year follow-up data were available, limiting interpretation of 3-year survival findings.

## Conclusion

Our findings underscore the high unmet need for improved treatments in patients with unresectable stage III NSCLC. Future studies differentiating patients by type of CRT regimen may provide further insight into how changes in clinical practice during the past two decades have affected survival in these patients. Knowledge of survival rates associated with historic standard therapies and how they have evolved over time also serves as an important starting point for understanding the potential benefits of new therapies and supporting health economic evaluations. It will, therefore, be important to revisit these analyses in future years, to examine the impact that more recently approved therapies may have had on OS.

## Data Availability

Data underlying the findings described in this manuscript may be obtained in accordance with AstraZeneca’s data sharing policy described at: https://astrazenecagrouptrials.pharmacm.com/ST/Submission/Disclosure.

## Abbreviations

AJCC: American Joint Committee on Cancer
CI: Confidence interval
CRT: Chemoradiotherapy
IQR: Interquartile range
K–M: Kaplan–Meie
NSCLC: Non-small cell lung cancer
OS: Overall survival
SEER: Surveillance, Epidemiology, and End Results

## References

[1] Novello S, Barlesi F, Califano R, Cufer T, Ekman S, Levra MG (2016). Metastatic non-small-cell lung cancer: ESMO clinical practice guidelines for diagnosis, treatment and follow-up. Ann Oncol.

[2] Chen VW, Ruiz BA, Hsieh MC, Wu XC, Ries LA, Lewis DR (2014). Analysis of stage and clinical/prognostic factors for lung cancer from SEER registries: AJCC staging and collaborative stage data collection system. Cancer.

[3] Patel V, Shrager JB (2005). Which patients with stage III non-small cell lung cancer should undergo surgical resection?. Oncologist.

[4] Surveillance, Epidemiology, and End Results Program. Cancer Stat Facts: Lung and Bronchus Cancer 2018. Available at: https://seer.cancer.gov/statfacts/html/lungb.html. Accessed 3 Dec 2018.

[5] Dillman RO, Seagren SL, Propert KJ, Guerra J, Eaton WL, Perry MC (1990). A randomized trial of induction chemotherapy plus high-dose radiation versus radiation alone in stage III non-small-cell lung cancer. N Engl J Med.

[6] Aupérin A, Le Péchoux C, Rolland E, Curran WJ, Furuse K, Fournel P (2010). Meta-analysis of concomitant versus sequential radiochemotherapy in locally advanced non-small-cell lung cancer. J Clin Oncol.

[7] Cheema PK, Rothenstein J, Melosky B, Brade A, Hirsh V (2019). Perspectives on treatment advances for stage III locally advanced unresectable non-small-cell lung cancer. Curr Oncol.

[8] Antonia SJ, Villegas A, Daniel D, Vicente D, Murakami S, Hui R (2017). Durvalumab after chemoradiotherapy in stage III non-small-cell lung cancer. N Engl J Med.

[9] Antonia SJ, Villegas A, Daniel D, Vicente D, Murakami S, Hui R (2018). Overall survival with durvalumab after chemoradiotherapy in stage III NSCLC. N Engl J Med.

[10] IMFINZI® (durvalumab). Prescribing Information. 2018. Available at: https://www.azpicentral.com/imfinzi/imfinzi.pdf#page=1. Accessed 27 Feb 2019.

[11] IMFINZI® (durvalumab). Summary of Product Characteristics. 2018 Available at: https://www.ema.europa.eu/en/documents/product-information/imfizi-epar-product-information_en.pdf. Accessed 15 May 2019.

[12] National Cancer Institute (NCI). Surveillance, Epidemiology, and End Results Program. SEER Fact Sheets: SEER Program Overview. Available at: https://seer.cancer.gov/about/overview.html. Accessed 17 Mar 2020.

[13] Schemper M, Smith TL (1996). A note on quantifying follow-up in studies of failure time. Control Clin Trials.

[14] Xia W, Yu X, Mao Q, Xia W, Wang A, Dong G (2017). Improvement of survival for non-small cell lung cancer over time. Onco Targets Ther.

[15] De Wever W, Coolen J, Verschakelen JA (2011). Imaging techniques in lung cancer. Breathe.

[16] Mac Manus MP, Wong K, Hicks RJ, Matthews JP, Wirth A, Ball DL (2002). Early mortality after radical radiotherapy for non-small-cell lung cancer: comparison of PET-staged and conventionally staged cohorts treated at a large tertiary referral center. Int J Rad Oncol Biol Phys.

[17] Gray JE, Villegas A, Daniel D, Vicente D, Murakami S (2020). Hui R, et al Three-year overall survival with durvalumab after chemoradiotherapy in Stage III NSCLC – update from PACIFIC. J Thorac Oncol.

